# Clinical factors associated with recent medical care visits in nursing homes: a multi-site cross-sectional study

**DOI:** 10.1186/s12877-022-03011-9

**Published:** 2022-04-12

**Authors:** Rebecca H. Correia, Fabrice I. Mowbray, Darly Dash, Paul R. Katz, Andrea Moser, Ryan P. Strum, Aaron Jones, Ahmad von Schlegell, Andrew P. Costa

**Affiliations:** 1 Department of Health Research Methods, Evidence, and Impact, McMaster University, Hamilton, ON Canada; 2 Department of Geriatrics, Florida State University College of Medicine, Tallahassee, FL USA; 3 Department of Family and Community Medicine, University of Toronto, Toronto, ON Canada; 4 Trillium Health Partners, Mississauga, ON Canada; 5 Schlegel Villages, Kitchener, ON Canada; 6 DeGroote School of Business, McMaster University, Hamilton, ON Canada

**Keywords:** Medical visits, Nursing home, Long-term care, Nursing home physician

## Abstract

**Objectives:**

We examined which resident-level clinical factors influence the provision of a recent medical care visit in nursing homes (NHs).

**Design:**

Multi-site cross-sectional.

**Setting and participants:**

We extracted data on 3,556 NH residents from 18 NH facilities in Ontario, Canada, who received at minimum, an admission and first-quarterly assessment with the Resident Assessment Instrument Minimum Data Set (MDS) 2.0 between November 1, 2009, and October 31, 2017.

**Methods:**

We conducted a secondary analysis of routinely collected MDS 2.0 data. The provision of a recent medical care visit by a physician (or authorized clinician) was assessed in the 14-day period preceding a resident’s first-quarterly MDS 2.0 assessment. We utilized best-subset multivariable logistic regression to model the adjusted associations between resident-level clinical factors and a recent medical care visit.

**Results:**

Two thousand eight hundred fifty nine (80.4%) NH residents had one or more medical care visits prior to their first-quarterly MDS 2.0 assessment. Six clinically relevant factors were identified to be associated with recent medical care visits in the final model: exhibiting wandering behaviours (OR = 1.34, 95% CI 1.09 – 1.63), presence of a pressure ulcer (OR = 1.37, 95% CI 1.05 – 1.78), a urinary tract infection (UTI) (OR = 1.52, 95% CI 1.06 – 2.18), end-stage disease (OR = 9.70, 95% CI 1.32 – 71.02), new medication use (OR = 1.31, 95% CI 1.09 – 1.57), and analgesic use (OR = 1.24, 95% CI 1.03 – 1.49).

**Conclusions and implications:**

Our findings suggest that resident-level clinical factors drive the provision of medical care visits following NH admission. Clinical factors associated with medical care visits align with the minimum competencies expected of physicians in NH practice, including managing safety risks, infections, medications, and death. Ensuring that NH physicians have opportunities to acquire and strengthen these competencies may be transformative to meet the ongoing needs of NH residents.

**Supplementary Information:**

The online version contains supplementary material available at 10.1186/s12877-022-03011-9.

## Introduction

The COVID-19 pandemic has highlighted the essential services provided within nursing homes (NHs) by multidisciplinary clinical teams [1]. While nursing staff and personal support workers provide the majority of bedside care, the complexity of NH residents warrants the need for physician care and mobile medical services [2–4]. In both Canada and the United States, a Most Responsible Provider (MRP) is assigned to each NH resident to provide routine medical assessments, ambulatory care visits, and determine clinical treatments and plans of care [5, 6]. MRPs exhibit knowledge, skills, and expertise in identifying and managing frailty, multimorbidity, polypharmacy, cognitive or behavioural conditions, and transitions of care [7].

Across North America, the frequency and pattern of NH resident visit schedules vary greatly based on current funding mandates [8]. In Ontario, Canada, MRPs provide routine services according to the Ontario Health Insurance Plan (OHIP) Fee Schedule, including admission and annual assessments, chronic disease management, monitoring changing health needs, ambulatory care visits, pre-procedural assessments, and palliative care services [6, 9]. Monthly Management fees account for the majority of physician claims in NHs, whereby MRPs are expected to provide a minimum of two assessments or progress notes, on average, per calendar month for routine medical care, management, or supervision of a NH resident [9]. In addition, NH physicians provide services such as engaging in shared-decision making with a resident’s family and care team, completing medication reviews, and participating in telephone consults [9]. The current Fee Schedule and mandates in Ontario do not strongly incentivize emergent and unscheduled medical care visits in NHs.

Prior work has attempted to make physician care in NHs more responsive to resident needs and complexity [10, 11], including same-day access to physician care [5]. However, little is known about what resident-level clinical factors influence medical care visits. Given that the vast majority of NH physicians in Ontario participate in a billing model that does not incentivize emergent and unscheduled medical care, it is an ideal sample to investigate the responsiveness of medical care to identifiable need. Our objective was to examine and determine what NH resident-level clinical factors influence the odds of receiving a medical care visit prior to a resident’s scheduled first-quarterly assessment.

## Methods

### Study design

We conducted a cross-sectional study of residents across a chain of 18 for-profit NHs in Ontario, Canada. Ethics approval was granted by the Hamilton Integrated Research Ethics Board (#2018–0739) for secondary analysis of the administrative and health data analyzed. We satisfied data security standards related to privacy and reporting, and we received a waiver of informed consent based on the absolute impractically of obtaining informed consent where the data were de-identified. The Strengthening the Reporting of Observational Studies in Epidemiology (STROBE) statement was used to report the results of this study [12].

### Data source

Data on residents’ clinical characteristics and outcomes were obtained from the Resident Assessment Instrument Minimum Data Set (MDS) 2.0 assessment. The MDS 2.0 is a comprehensive and standardized clinical assessment instrument that examines over 300 items from 15 health domains, including physical and cognitive functioning, medical diagnoses, social support, medication use, and health service use [13–15]. MDS 2.0 assessments were conducted by trained NH nursing staff as the standard practice [16].

The majority of Canadian provinces and territories use the MDS 2.0, or its successor, the interRAI Long-Term Care Facilities (LTCF) instrument, to assess all NH residents at admission and quarterly thereafter, until death or discharge [17]. The MDS is also mandated for use in the United States; however, the MDS 3.0 version is most commonly used [18]. Data for this study were obtained from an anonymized, population-level health administrative database.

### Setting and participants

Our cohort comprised NH residents admitted to any facility within a large for-profit chain who received a minimum of two routine MDS 2.0 assessments (i.e., on intake to facility and first-quarterly) between November 1, 2009, and October 31, 2017. All residents were included in our analysis, regardless of pre-existing health conditions, resident complexity, or clinical factors. To examine the relationship between resident health and MRP encounters, we examined each NH resident’s first-quarterly MDS 2.0 assessment record which is routinely conducted 90 days after admission to a NH. The first-quarterly MDS 2.0 assessment was of interest to rule out the additional variance introduced by greater health instability and increased emergency department (ED) use during the transitory admission period [19].

### Variables and measurement

Recent medical care visits in NHs may be impacted by many clinical factors related to the resident’s complexity or medical acuity. A total of 42 independent measures (see Additional file 1) that are routinely collected in MDS 2.0 assessments were selected as candidate predictors *a priori*, based on their relevance to newly developed primary care provider quality indicators for NH care and The Society for Post-Acute and Long-Term Care Medicine (AMDA) physician competency domains [20, 21].

We examined demographic (age and sex) and a series of clinical factors including, but not limited to: cognition, mood, and behaviour; falls, mobility, and pressure ulcers; pain management; urinary; medication use; special treatments and procedures; and clinical symptoms. Unless otherwise mentioned, all variables were left in their original measurement form (see Additional file 1).

### Outcome measure

The primary outcome was a documented medical care visit by a physician (or authorized assistant or practitioner) in the 14 days preceding a resident’s first-quarterly MDS 2.0 assessment. Medical care visits were defined as partial or full examinations at the NH facility or in the physician’s office – excluding exams conducted in the ED [13]. Medical care visits included those conducted by medical doctors, osteopaths, podiatrists, or dental experts, who served as either the primary or consulting clinician. Authorized physician assistants and nurse practitioners working in collaboration were also included. The availability of data captured in the MDS 2.0 restricted our timeframe of analysis to medical care visits occurring 14 days prior to the first-quarterly assessment.

### Statistical analysis

Each resident acted as a single unit of analysis. General frequency and central tendency measures were computed to provide descriptive statistics. Univariable and multivariable logistic regression was employed to identify the factors associated with one or more recent medical care visits in NHs. A series of univariable logistic regression models were computed to determine the observed relationship between each clinical factor of interest and recent medical care visits. Best-subset multivariable logistic regression was used to identify the subset of clinical covariates that best defined the relationship with recent medical care visits. The Akaike information criterion (AIC) was used to evaluate model fit. A best-subset of clinically relevant factors were selected to avoid suppressor effects commonly found with stepwise methods and statistically pre-determined predictors (e.g., *p *< 0.25) [22]. Cases with missing data were deleted within each analysis. All analyses were performed using Statistical Analysis Software (SAS), version 9.2 (SAS Institute, Inc., Cary, North Carolina). Our model, using all candidate predictors, had an event-per-variable ratio > 10, as recommended to improve the external validity of findings [22].

## Results

We identified 3,556 NH residents who had a first-quarterly MDS 2.0 assessment completed between November 15, 2009, and October 20, 2017 (Table 1). Residents were of advanced age (median 85 years, IQR = 10.8) and predominantly female (62.0%). The majority of residents had moderate or severe cognitive impairments (53.5%), and many were diagnosed with Alzheimer’s disease (18.3%) or a different taxonomy of dementia (54.5%). Most residents exhibited health stability, whereby very few experienced acute episodes or flare-ups of recurrent or chronic problems (7.8%) or end-stage disease (1.3%). Some residents had an ED visit (24.3%) or an inpatient hospital stay (23.6%) before their first-quarterly assessment. Overall, 2,859 (80.4%) NH residents had one or more medical care visits in the 14 days before their first-quarterly MDS 2.0 assessment.

**Table 1:** Characteristics of study participants (*N* = 3,556)

**Variable**	n (%)
**Demographics**	
**Age** (Years) ^**a**^	85 (10.8)
**Sex** (Female)	2,206 (62.6)
**Cognition, mood, behaviour**	
**Diagnosis** Alzheimer’s disease Dementia other than Alzheimer’s disease ^b^ Anxiety disorder or depression	551 (18.3) 1,939 (54.5) 887 (24.9)
**Moderately or severely impaired cognitive skills for daily decision-making** ^c^	1,901 (53.5)
**Deteriorated cognitive status** ^d^	197 (5.5)
**Wandering behaviours exhibited**^e^	1,085 (30.5)
**Presence of ≥ 1 indicators of delirium** ^c^	2,542 (71.5)
**Parenteral/IV or feeding tube**^**c**^	64 (1.8)
**Mood** ≥ 1 indicators of depression, sadness, or anxiety ^c^ Deteriorated mood ^d^	2,096 (58.9) 251 (7.1)
**Falls, mobility, pressure ulcers**	
**Fall occurred in last 180 days**	1,619 (45.5)
**Fractures** Hip fracture occurred in last 180 days Other fracture occurred in last 180 days	71 (2.0) 73 (2.1)
**Presence of ≥ 1 ulcers** (Any stage) ^c^	571 (16.1)
**Hypotension**	44 (1.5)
**Pain management**	
**Frequency** (Daily or less than daily) ^c f^	1,243 (35.0)
**Intensity** (Moderate, horrible, or excruciating) ^c f^	2,888 (81.2)
**Urinary**	
**Incontinence** Bladder ^g h^ Bowel ^g h^ Deteriorated urinary continence^d^	2,647 (74.4) 2,011 (56.6) 234 (6.6)
**Urinary tract infection**^§§^	302 (8.5)
**Ostomy present **^g^	52 (1.5)
**Urinary catheter present** (External, indwelling, or intermittent) ^g^	144 (4.05)
**Clinical symptoms**	
**Stability of conditions** Acute episode or flare-up of recurrent or chronic problem^c^ End-stage disease^e^^||^	276 (7.8) 46 (1.3)
**Chronic diseases** Diabetes mellitus Congestive heart failure Hypertension Stroke COPD Cancer	885 (24.9) 347 (11.6) 1,951 (64.9) 763 (21.5) 401 (13.3) 403 (13.4)
**Weight gain or loss of ≥ 1.5 kg** (3 lbs.) ^c^	20 (0.6)
**Shortness of breath** ^c^	262 (7.4)
**Medications**	
**New medications initiated** ^d^	1,909 (63.5)
**Number of medications** ^a^ ^c^	10 (5.0)
**Received the following medications for ≥ 1 days** ^c^ Psychoactive medication (Antipsychotic, antianxiety, antidepressant, or hypnotic) Diuretic Analgesic	2,436 (68.5) 1,010 (28.4) 2,170 (61.0)
**Special treatments and procedures**	
**Past hospital use** ^d^ ≥ 1 admissions with an overnight stay ≥ 1 ED visits	839 (23.6) 863 (24.3)

*IV* Intravenous Therapy, *COPD* Chronic Obstructive Pulmonary Disease, *ED* Emergency Department

^a^ Reported as median (Interquartile range)

^b^ Includes diagnoses of organic brain syndrome or chronic brain syndrome, senility, senile dementia, multi-infarct dementia, and dementia related to neurologic diseases other than Alzheimer’s (e.g., Picks, Creutzfeld-Jacob, Huntington’s disease, etc.)

^c^ Observation period: last 7 days

^d^ Observation period: last 90 days

^e^ Behaviour exhibited at any frequency in the last 7 days (e.g., 1 to 3 days, 4 to 6 days, or daily)

^f^ Any type of physical pain or discomfort in any part of the body. Pain may be localized to one area, or may be more generalized. It may be acute or chronic, continuous, or intermittent, or occur at rest or with movement

^g^ Observation period: last 14 days

^h^ Incontinent episodes occurring once a week or less (bladder) or less than weekly (bowel), occasionally incontinent, frequently incontinent, or having inadequate control

^§§^ Observation period: last 30 days

^||^ Six months or less to live

### Resident clinical factors associated with recent medical care visits

Unadjusted and adjusted odds ratios can be found in Table 2. The best multivariable model for the relationship between recent medical care visits and NH residents’ clinical factors included seven covariates. Statistically significant odds ratios were observed for six factors entered in the final model: exhibiting wandering behaviours (OR = 1.34, 95% CI 1.09 – 1.63), the presence of a pressure ulcer (OR = 1.37, 95% CI 1.05 – 1.78), a current urinary tract infection (UTI) (OR = 1.52, 95% CI 1.06 – 2.18), end-stage disease (OR = 9.70, 95% CI 1.32 – 71.02), starting new medications (OR = 1.31, 95% CI 1.09 – 1.57), and analgesic use (OR = 1.24, 95% CI 1.03 – 1.49). Statistical adjustment did not influence the statistical significance of these predictors, highlighting the robustness of these associations. The multivariable model achieved good fit using the Hosmer and Lemeshow goodness-of-fit test (***x***^***2***^=6.61, *df* = 7, *p* = 0.47).

**Table 2:** Clinical factors associated with medical care visits

Variable	Unadjusted Analysis		Adjusted Analysis
	**OR (95% CI)**		**aOR (95% CI)**
**Demographics**			
**Age** (Years)	1.00 (0.99 – 1.01)	-	
**Sex** (Female)	0.87 (0.73 – 1.03)	-	
**Cognition, mood, behaviour**			
**Diagnosis** Alzheimer’s disease Dementia other than Alzheimer’s disease ^b^ Anxiety disorder or depression	0.90 (0.72 – 1.12) 1.09 (0.92 – 1.29) 1.06 (0.87 – 1.28)	- - -	
**Moderately or severely impaired cognitive skills for daily decision-making** ^c^	1.09 (0.92 – 1.28)	-	
**Deteriorated cognitive status** ^d^	1.50 (1.00 – 2.26)	-	
**Wandering behaviours exhibited**^e^	1.23 (1.02 – 1.48) ^a^	1.34 (1.09 – 1.63) ^a^	
**Presence of ≥ 1 indicators of delirium** ^c^	0.94 (0.78 – 1.13)	-	
**Parenteral/IV or feeding tube**^c^	5.04 (1.58 – 16.1) ^a^	-	
**Mood** ≥ 1 indicators of depression, sadness, or anxiety ^c^ Deteriorated mood ^d^	1.21 (1.02 – 1.43) ^a^ 1.16 (0.83 – 1.62)	- -	
**Falls, mobility, pressure ulcers**			
**Fall occurred in last 180 days**	1.25 (1.05 – 1.47) ^a^	-	
**Fractures** Hip fracture occurred in last 180 days Other fracture occurred in last 180 days	2.68 (1.16 – 6.21) ^a^ 1.03 (0.57 – 1.85)	2.19 (0.93 – 5.12) -	
**Presence of ≥ 1 ulcers** (Any stage) ^c^	1.36 (1.07 – 1.74) ^a^	1.37 (1.05 – 1.78) ^a^	
**Hypotension**	1.01 (0.48 – 2.12)	-	
**Pain management**			
**Frequency** (Daily or less than daily) ^c f^	1.33 (1.12 – 1.60) ^a^	-	
**Intensity** (Moderate, horrible, or excruciating) ^c f^	0.81 (0.65 – 1.02)	-	
**Urinary**			
**Incontinence** Bladder ^g h^ Bowel ^g h^ Deteriorated urinary continence^d^	1.19 (0.99 – 1.43) 1.22 (1.03 – 1.44) ^a^ 0.89 (0.64 – 1.23)	- - -	
**Urinary tract infection**^**§§**^	1.66 (1.18 – 2.34) ^a^	1.52 (1.06 – 2.18) ^a^	
**Ostomy present **^g^	0.73 (0.39 – 1.37)	-	
**Urinary catheter present** (External, indwelling, or intermittent) ^g^	1.86 (1.11 – 3.11) ^a^	-	
**Clinical symptoms**			
**Stability of conditions** Acute episode or flare-up of recurrent or chronic problem ^c^ End-stage disease ^e ||^	1.48 (1.05 – 2.09) ^a^ 11.13 (1.53 – 80.91)^a^	- 9.70 (1.32 – 71.02) ^a^	
**Chronic diseases** Diabetes mellitus Congestive heart failure Hypertension Stroke COPD Cancer	0.92 (0.76 – 1.12) 1.22 (0.92 – 1.64) 0.99 (0.83 – 1.20) 1.10 (0.89 – 1.35) 0.92 (0.71 – 1.18) 0.97 (0.75 – 1.26)	- - - - - -	
**Weight gain or loss of ≥ 1.5 kg** (3 lbs.) ^c^	4.66 (0.62 – 34.85)	-	
**Shortness of breath** ^c^	1.47 (1.03 – 2.10) ^a^	-	
**Medications**			
**New medications initiated** ^d^	1.42 (1.18–1.69) ^a^	1.31 (1.09 – 1.57) ^a^	
**Number of medications** ^c^	1.02 (1.00 – 1.04)	-	
**Received the following medications for ≥ 1 days** ^c^ Psychoactive medication (Antipsychotic, antianxiety, antidepressant, or hypnotic) Diuretic Analgesic	1.03 (0.86 – 1.23) 1.18 (0.97 – 1.42) 1.36 (1.15 – 1.60) ^a^	- - 1.24 (1.03 – 1.49) ^a^	
**Special treatments and procedures**			
**Past hospital use** ^d^ ≥ 1 admissions with an overnight stay ≥ 1 ED visits	1.56 (1.26 – 1.93) ^a^ 1.61 (1.30 – 1.99) ^a^	- -	

*OR* Odds Ratio, *aOR* Adjusted Odds Ratio, *CI* Confidence Interval, *IV* Intravenous Therapy, *COPD* Chronic Obstructive Pulmonary Disease, *ED* Emergency Department

^a^ Significant at the level of .05

^b^ Includes diagnoses of organic brain syndrome or chronic brain syndrome, senility, senile dementia, multi-infarct dementia, and dementia related to neurologic diseases other than Alzheimer’s (e.g., Picks, Creutzfeld-Jacob, Huntington’s disease, etc.)

^c^ Observation period: last 7 days

^d^ Observation period: last 90 days

^e^ Behaviour exhibited at any frequency in the last 7 days (e.g., 1 to 3 days, 4 to 6 days, or daily)

^f^ Any type of physical pain or discomfort in any part of the body. Pain may be localized to one area, or may be more generalized. It may be acute or chronic, continuous, or intermittent, or occur at rest or with movement

^g^ Observation period: last 14 days

^h^ Incontinent episodes occurring once a week or less (bladder) or less than weekly (bowel), occasionally incontinent, frequently incontinent, or having inadequate control

^§§^ Observation period: last 30 days

^||^ Six months or less to live

### Missing data

On average, 16.5% of data were missing for the 42 independent measures (*n* = 588 residents were missing at least one data point), while none were missing for the outcome.

## Discussion

### Main findings

Most NH residents received a recent medical care visit preceding their first-quarterly MDS 2.0 assessment, affirming the need for medical care visits and support during the transitionary NH admission period. NH medical care may be impacted by many resident-level factors, including resident demographics, medical complexity, and lability of clinical symptoms [10, 23, 24]. We identified six clinically important factors that increased the odds of a recent medical care visit in newly admitted NH residents, including wandering, pressure ulcers, UTIs, end-stage disease, new medication use, and analgesic use. In the context of a predominantly scheduled billing model, we found that medical care visits are sensitive to resident needs aligned with the minimum competencies expected of physicians in NH practice.

A minimum expected competency of NH physicians includes having the knowledge and skill to identify when to implement interventions as to minimize risk factors and optimize resident safety [21]. The adverse consequences of wandering – including falls and other accidents, getting lost, malnutrition, fatigue, sleep disturbance, social isolation, kinship issues, and injury, including fractures – may necessitate medical care visits [25–27]. Pressure ulcers are associated with considerable mortality, distress, and discomfort among residents, all of which may drive medical care visits [28]. However, we could not discern whether medical care visits were specifically related to assessing or managing pressure ulcers, or if NH residents with pressure ulcers were more likely to have other acute health changes that drive medical care visits. Due to a lack of consensus on the diagnostic criteria for UTIs in older adults, stemming from inconsistent symptomology and biomarkers, the identification and treatment often varies across NH facilities and between residents [29–31]. In turn, additional physician time and resources may be required to properly diagnose and treat UTIs among NH residents with atypical or complex presentations, which may drive medical care visits. The reliance on antibiotic therapy for NH residents with UTIs may also influence visitation because of the high rates of adverse effects and poor tolerance for antibiotics [30, 31].

End-stage disease is one of several indicators of the MDS-Changes in Health, End-stage Disease and Symptoms and Signs (CHESS) score, a valid predictor of mortality, and is associated with greater physician involvement and use of medical treatments [32]. Medically unstable residents often have abnormal laboratory values and responsive changes in physician orders – both of which may influence medical care visits [32]. NH physicians are also expected to identify circumstances where palliative and end-of-life services may benefit the resident and family. The presence of, or progression to, end-stage disease may warrant further attention of physicians to support shared-decision making and dignity in death [21]. AMDA sets the minimum standard for NH physicians to prescribe and adjust medications prudently and evaluate resident adverse events and outcomes resulting from medication errors [21]. Older adults metabolize and respond to medications differently than their younger counterparts, and often require closer monitoring to ensure adequate therapeutic management and minimal adverse side effects [33, 34]. The high rates of polypharmacy in older adults further increases the risk of drug interactions [35]. Lastly, physicians often report challenges in recognizing and treating pain, especially for residents with cognitive impairments [36–38]. Interestingly, two independent measures, pain frequency and intensity, were highly prevalent in our sample but were not included in the final multivariable model. This finding aligns with prior work in that increased physician care is associated with reporting pain and being prescribed analgesics, whereas unreported pain is typically managed without medications [38].

### Clinical and policy implications

In our sample, one-fifth of NH residents did not receive any medical care visits by physicians within two weeks of their first-quarterly assessment. In NHs, older adults are typically frail with multimorbidity, complex health needs, and limited ability to seek medical services outside of NH facilities [6, 23]. Consistent follow-up is crucial to ensure that NH residents are monitored and that their health concerns are managed proactively [39]. Additionally, physician competence and knowledge of the clinical factors that drive medical care visits – namely, safety risks, infections, medications, and death – are essential to support the identification and management of NH residents. COVID-19 illustrated the consequences associated with fewer medical care visits [40]. During the first wave of COVID-19 in Ontario, fewer medical care visits and care orders occurred in NHs, suggesting that in-person medical care visits were not replaced with virtual visits [40]. Our findings suggest that NH resident needs drive recent medical care visits, and supporting physician competence in regard to these clinical factors may promote the prognosis and quality of care of NH residents.

### Strengths and limitations

Our study is unique in identifying resident-level clinical factors that are significantly associated with medical care visits across a large chain of NHs. The demographic and clinical profile of this cohort is similar to the broader population of NH residents in Ontario, supporting the generalizability of our results [41]. This analysis included NH facilities in a single chain, allowing for structural variables to be held constant (e.g., size, staff organization, physician visit and fee schedules) – strengthening the internal validity of our findings. Candidate predictor selection was guided by NH physician quality indicators and competency domains, allowing for the evaluation of informative assessment items concerning the frequency and need for medical care visitation in NHs. However, this study is not without its limitations.

While the study’s large sample size allowed for control of a broad range of clinical factors, some potential confounders were not available in the dataset and could not be controlled for. Similarly, the quality of the medical care visit was unknown and characteristics that may have been pertinent were not examined (e.g., whether the visit occurred in-person or over the phone, whether a physical assessment occurred, the type of clinician who conducted the visit, and time spent during the encounter). We were unable to differentiate in-person medical care visits associated with the Monthly Management Fee Schedule, from those occurring in response to emergent medical needs or conditions. However, the associations between medical care visits and measures of medical complexity suggest that in-person visits are largely in response to resident needs. If many of the in-person visits were routine, then that would bias in favour of no association; therefore, our associations are likely conservative. Additionally, we were unaware of whether residents received medical care visits outside the 14 days prior to their first-quarterly assessment. However, the timing in which residents received their quarterly assessment varied greatly (see Additional file 2), mitigating this risk of bias. While the 14-day timeframe specified in the MDS 2.0 may be perceived as arbitrary, this specific and measurable period reduces the potential for recall bias and makes our association more conservative.

Additionally, while pairwise deletion maximizes available data for our analysis and increases power, it assumes that all data are missing completely at random [42]. We identified that 16.5% of all data points were missing across six missing data patterns. Data may be missing because some variables are only collected in “full” versions of the MDS 2.0, which is only required at entry to NHs, annual assessments, or if significant clinical changes occur; other times, the shorter MDS 2.0 is completed. Since best-subset regression tends to overfit the data, we considered only clinically relevant candidate predictors and gave additional consideration to whether variables with a low prevalence in the sample (e.g., parenteral/IV or feeding tube and end-stage disease) were included in the multivariable model. We conducted a sensitivity analysis without end-stage disease in the final model, and the interpretations of the other variables in the multivariable model did not change (see Additional file 3).

## Conclusions and implications

We identified six resident-level clinical factors that were significantly associated with recent medical care visits in NHs. NH residents exhibiting wandering behaviours, having one or more ulcers of any stage, a UTI, end-stage disease, starting new medications, or using analgesics had greater odds of receiving medical care visits. These clinical factors suggest that physician practice in NHs corresponds to competence among NH physicians to manage safety risks, infections, medications, and death. Ensuring NH physicians have opportunities to acquire and strengthen these competencies may be transformative to meet the ongoing needs of NH residents.

## Conflicts of interest

This project received no formal funding, and the authors have no financial or personal conflicts of interest to disclose.

## Supplementary Information

**Additional file 1:** **Table S1.** Definitions and measurement of independent variables.

**Additional file 2:** **Figure S1.** Distribution of days between admission and first-quarterly MDS 2.0 assessment.

**Additional file 3:** **Table S2.** Clinical factors associated with medical care visits without end-stage disease in the final model (sensitivity analysis).

## Data Availability

The datasets analyzed during the current study are not publicly available due to containing personal health information. The data were used under license for the current study and so are not publicly available. The corresponding author can be contacted regarding any requests for the data from this study.

## Abbreviations

NH: Nursing home
NHs: Nursing homes
MRP: Most Responsible Provider
OHIP: Ontario Health Insurance Plan
STROBE: Strengthening the Reporting of Observational Studies in Epidemiology
MDS: Minimum Data Set
LTCF: Long-Term Care Facilities
ED: Emergency department
AMDA: The Society for Post-Acute and Long-Term Care Medicine
AIC: Akaike information criterion
SAS: Statistical Analysis Software
OR: Odds ratio
CI: Confidence interval
UTI: Urinary tract infection
CHESS: Changes in Health, End-stage Disease and Symptoms and Signs
