# Supplementary material for: Clinical factors associated with recent medical care visits in nursing homes: a multi-site cross-sectional study
Source: BMC Geriatr. 2022 Apr 12;22:320. doi: 10.1186/s12877-022-03011-9 (PMC9003172; doi:10.1186/s12877-022-03011-9)
Supplement: Supplementary file 1 — Additional file 1: Table S1. Definitions and measurement of independent variables. [file 12877_2022_3011_MOESM1_ESM.docx]

**Additional file 1**

**Table S1. Definitions and measurement of independent variables**

|  | **Variable type** |
| --- | --- |
| **Demographics** | |
| 1. Age | Continuous |
| 1. Sex | Categorical (2) |
| **Cognition, mood, and behaviour** | |
| 1. Diagnosed with Alzheimer’s disease | Categorical (2) |
| 1. Diagnosed with dementia other than Alzheimer’s disease | Categorical (2) |
| 1. Diagnosed anxiety disorder or depression (either or both) | Categorical (2) |
| 1. Cognitive skills for daily decision-making in the last 7 days (i.e., moderate/severe impairment compared to independent/modified independence) | Categorical (2) |
| 1. Deteriorated cognitive status, skills, or abilities compared to no change or improvement 90 days ago | Categorical (2) |
| 1. Exhibited wandering behaviours (i.e., moving with no rational purpose, seemingly oblivious to needs or safety) at least once in the last 7 days | Categorical (2) |
| 1. Exhibited indicators of delirium (i.e., periodic disordered thinking or awareness) at least once in the last 7 days | Categorical (2) |
| 1. Required a parenteral/intravenous (IV) or feeding tube in the last 7 days | Categorical (2) |
| 1. Persistence of one or more indicators of depression, sadness, or anxiety in the last 7 days | Categorical (2) |
| 1. Deteriorated mood compared to no change or improvement 90 days ago | Categorical (2) |
| **Falls, mobility, and pressure ulcers** | |
| 1. Experienced a fall in the last 180 days | Categorical (2) |
| 1. Experienced a hip fracture in the last 180 days | Categorical (2) |
| 1. Experienced any other fracture in the last 180 days | Categorical (2) |
| 1. Presence of ulcers of any stage in the last 7 days | Categorical (2) |
| 1. Diagnosed with hypotension | Categorical (2) |
| **Pain management** | |
| 1. Any self-reported physical pain or discomfort occurring daily or less than daily in the last 7 days (i.e., pain frequency) | Categorical (2) |
| 1. Moderate, horrible, or excruciating pain, as compared to mild pain (i.e., pain intensity) | Categorical (2) |
| **Urinary** | |
| 1. Experienced at least one bladder incontinent episode in the last 7 days, compared to complete control | Categorical (2) |
| 1. Experienced at least one bowel incontinent episode in the last 7 days, compared to complete control | Categorical (2) |
| 1. Deteriorated urinary continence in the last 90 days compared to no change or improvement | Categorical (2) |
| 1. Presence of a urinary tract infection (UTI) in the last 30 days | Categorical (2) |
| 1. Presence of an ostomy | Categorical (2) |
| 1. Presence of a urinary catheter (e.g., external, indwelling, or intermittent) in the last 14 days | Categorical (2) |
| **Clinical symptoms** | |
| 1. Occurrence of an acute episode or flare-up of a current or chronic problem | Categorical (2) |
| 1. Presence of an end-stage disease (i.e., six months or less to live) | Categorical (2) |
| 1. Diagnosed with diabetes mellitus | Categorical (2) |
| 1. Diagnosed with congestive heart failure (CHF) | Categorical (2) |
| 1. Diagnosed with hypertension | Categorical (2) |
| 1. Diagnosed with stroke | Categorical (2) |
| 1. Diagnosed with chronic obstructive pulmonary disease (COPD) | Categorical (2) |
| 1. Diagnosed with cancer | Categorical (2) |
| 1. Experienced weight gain or loss of 1.5 or more kilograms (3 lbs.) in the last 7 days | Categorical (2) |
| 1. Experienced shortness of breath | Categorical (2) |
| **Medication use** | |
| 1. New medication initiated and used in the last 7 days | Categorical (2) |
| 1. Number of medications taken | Continuous |
| 1. Use of any psychoactive (i.e., antipsychotic, antianxiety, antidepressant, or hypnotic) medication in the last 7 days | Categorical (2) |
| 1. Use of a diuretic in the last 7 days | Categorical (2) |
| 1. Use of an analgesic medication in the last 7 days | Categorical (2) |
| **Special treatments and procedures** | |
| 1. Admission to hospital for an overnight stay in the last 90 days | Categorical (2) |
| 1. Emergency department (ED) visit in the last 90 days | Categorical (2) |
