# Supplementary material for: Clinical factors associated with recent medical care visits in nursing homes: a multi-site cross-sectional study
Source: BMC Geriatr. 2022 Apr 12;22:320. doi: 10.1186/s12877-022-03011-9 (PMC9003172; doi:10.1186/s12877-022-03011-9)
Supplement: Supplementary file 2 — Additional file 2: Figure S1. Distribution of days between admission and first-quarterly MDS 2.0 assessment. [file 12877_2022_3011_MOESM2_ESM.docx]

**Additional file 2**

**Figure S1. Distribution of days between admission and first-quarterly MDS 2.0 assessment**
