# Supplementary material for: Clinical factors associated with recent medical care visits in nursing homes: a multi-site cross-sectional study
Source: BMC Geriatr. 2022 Apr 12;22:320. doi: 10.1186/s12877-022-03011-9 (PMC9003172; doi:10.1186/s12877-022-03011-9)
Supplement: Supplementary file 3 — Additional file 3: Table S2. Clinical factors associated with medical care visits without end-stage disease in the final model (sensitivity analysis). [file 12877_2022_3011_MOESM3_ESM.docx]

**Additional file 3**

**Table S2. Clinical factors associated with medical care visits without end-stage disease in the final model** (sensitivity analysis)

| **Variable** | **Adjusted Analysis** |
| --- | --- |
|  | **aOR (95% CI)** |
| **Cognition, mood, behaviour** | |
| **Wandering behaviours exhibited** † | 1.32 (1.08 – 1.61) * |
| **Falls, mobility, pressure ulcers** | |
| **Fractures**  Hip fracture in last 180 days | 2.14 (0.92 – 5.02) |
| **Presence of ≥1 ulcers** (any stage) ^‡^ | 1.41 (1.09 – 1.83) * |
| **Urinary** | |
| **Urinary tract infection** ^§^ | 1.52 (1.06 – 2.17) * |
| **Medications** | |
| **New medications initiated** ^\|\|^ | 1.31 (1.10 – 1.58) * |
| **Received the following medications for ≥1 days** ^‡^  Analgesic | 1.24 (1.04 – 1.49) * |
| OR=Odds Ratio; aOR=Adjusted Odds Ratio; CI=Confidence Interval  * Significant at the level of .05  ^†^ Behaviour exhibited at any frequency in the last 7 days (e.g., 1 to 3 days, 4 to 6 days, or daily  ^‡^ Observation period: last 7 days  ^§^ Observation period: last 30 days  ^\|\|^ Observation period: last 90 days | |
